# Rv2299c, a novel dendritic cell-activating antigen of *Mycobacterium tuberculosis*, fused-ESAT-6 subunit vaccine confers improved and durable protection against the hypervirulent strain HN878 in mice

**DOI:** 10.18632/oncotarget.15256

**Published:** 2017-02-10

**Authors:** Han-Gyu Choi, Seunga Choi, Yong Woo Back, Seungwha Paik, Hye-Soo Park, Woo Sik Kim, Hongmin Kim, Seung Bin Cha, Chul Hee Choi, Sung Jae Shin, Hwa-Jung Kim

**Affiliations:** ^1^ Department of Microbiology, and Medical Science, College of Medicine, Chungnam National University, Daejeon, Republic of Korea; ^2^ Department of Microbiology, Institute for Immunology and Immunological Diseases, Brain Korea 21 PLUS Project for Medical Science, Yonsei University College of Medicine, Seoul, Republic of Korea

**Keywords:** tuberculosis, DC maturation, toll-like receptor 4, multifunctional T cell, subunit vaccine

## Abstract

Understanding functional interactions between DCs and antigens is necessary for achieving an optimal and desired immune response during vaccine development. Here, we identified and characterized protein Rv2299c (heat-shock protein 90 family), which effectively induced DC maturation. The Rv2299c-maturated DCs showed increased expression of surface molecules and production of proinflammatory cytokines. Rv2299c induced these effects by binding to TLR4 and stimulating the downstream MyD88-, MAPK- and NF-κB-dependent signaling pathways. The Rv2299c-maturated DCs also showed an induced Th1 cell response with bactericidal activity and expansion of effector/memory T cells. The Rv2299c-ESAT-6 fused protein had greater immunoreactivity than ESAT-6. Furthermore, boosting BCG with the fused protein significantly reduced hypervirulent *Mycobacterium tuberculosis* HN878 burdens post-challenge. The pathological study of the lung from the challenged mice assured the efficacy of the fused protein. The fused protein boosting also induced Rv2299c-ESAT-6-specific multifunctional CD4^+^ T-cell response in the lungs of the challenged mice. Our findings suggest that Rv2299c is an excellent candidate for the rational design of an effective multiantigenic TB vaccine.

## INTRODUCTION

*Mycobacterium tuberculosis* (Mtb) is one of the most successful human pathogens, with one-third of the world's population being infected [1]. Because the only available vaccine, *M. bovis* Bacillus Calmette Guerin (BCG), is unable to provide significant protection against tuberculosis (TB) in adults [2], a more effective vaccine for replacing or boosting BCG is clearly needed. Currently, one of the reigning strategies in TB vaccine research is to develop BCG-booster vaccines using adjuvanted protein subunits. These heterologous prime-boost strategies have proven a powerful mode of vaccination. It is important to identify and characterize the mycobacterial antigens involved in the induction of protective immunity for effective development of prospective TB vaccine candidates. However, there are few antigens that have been used in preparation of TB vaccines that are currently in various phases of clinical trials [3].

Th1 immune responses are essential for controlling Mtb infection. Disruption of genes involved with Th1-related cytokines such as IFN-γ and IL-12 increases the susceptibility to mycobacterial infection in mice and humans [4]. Therefore, many studies on TB vaccines have been focused on strong T-cell-stimulating antigens, such as antigen 85 complex (Ag85) and ESAT-6 [5]. T-cell responses, which are essential for controlling infection, rarely eliminate Mtb from infected humans or animals [6–8]. Although strong T-cell-stimulating antigens induce robust protective immunity in mice, these antigens cannot induce complete sterilizing immunity [9, 10].

Dendritic cells (DCs), the most professional antigen-presenting cells in the immune system, are key players involved in bridging the innate and adaptive immunity. It has been suggested that Mtb subverts CD4 T-cell-dependent immunity by delaying initiation of T-cell responses via modulation of DC functions [11–14] and survives in a dormant form. Therefore, early activation and migration of DCs to draining lymph nodes together with stimulation of T cells are key factors for inducing effective protection against Mtb infection. These observations suggest that a mycobacterial antigen that elicits effective host protective immunity via DC activation is a promising target for development of a TB vaccine. In fact, DCs infected with BCG or pulsed with Mtb antigens induce significant protection to a challenge with both moderate and high doses of virulent Mtb in a mouse model [15, 16]. Although several mycobacterial proteins that activate DCs to drive a Th1 immune response have been identified, little is known about their detailed antimycobacterial mechanism and about protective efficacy of the protein itself as a vaccine.

ESAT-6-containing vaccines such as H1 or H56 have been demonstrated to confer efficient protection against Mtb H37Rv in pre- or post-exposure animal models, and the fusion protein is more protective than either component [10, 17]. Here, we hypothesized that incorporating DC-activating protein would improve long-term efficacy of the vaccine containing only T-cell antigens. Because DCs maturated by a DC-activating protein are an effective antigen-presenting cell for generation of a long-term Th1 memory response against a T-cell-stimulating antigen, and the DC-activating protein itself can strongly drive Th1 polarization. It has been reported that mycobacterial heat-shock proteins (HSPs) including HSP65 induce strong protective immunity against TB [18]. In this study, we identified the Rv2299c protein (belongs to the HSP90 family), which effectively induced DC maturation, and then we analyzed its antimycobacterial mechanism through DC activation to elicit strong Th1-type responses. Next, we tested protective vaccine efficacy of the Rv2299c protein or Rv2299c-fused ESAT-6 protein against Mtb HN878 clinical isolates. Our results suggest that Rv2299c-maturated DCs induce a Th1 cell response with antimycobacterial activity, and the fusion protein consisting of Rv2299c and ESAT-6—as a new concept of a DC-activating protein-based vaccine—is a promising way of boosting BCG.

## RESULTS

### The recombinant Rv2299c protein induces maturation and activation of DCs

**Figure 1:**
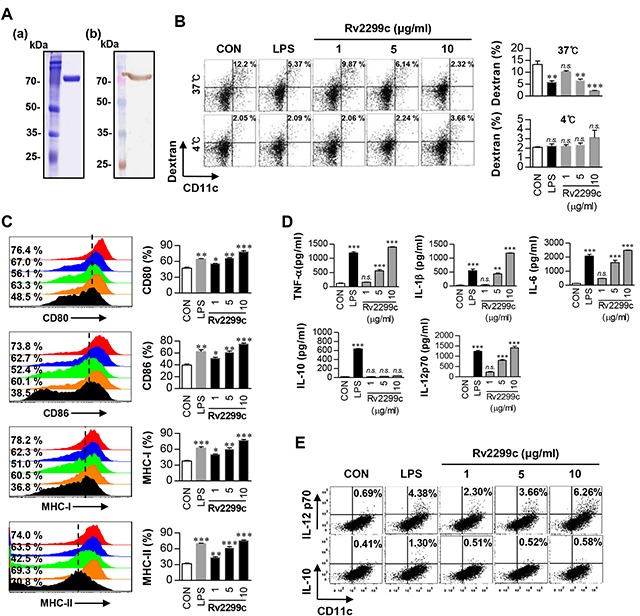
Recombinant Rv2299c induces DC maturation **A**. Purified recombinant Rv2299c protein was analyzed by SDS-PAGE with Coomassie blue staining (a) and western blot analysis using anti-His antibodies (b). DCs were activated with the indicated concentration of Rv2299c or LPS (100 ng/ml) for 24 hr. **B**. Activated DCs were incubated with dextran-FITC at 37°C or 4°C for 30 min and assessed by FACS analysis of dextran (FITC) uptake. The percentages of dextran (FITC)-positive CD11c (PE)-positive cells are indicated. The results are representative of the results of four experiments. Bar graphs show mean ± SEM of four experiments. **C**. Activated DCs were stained with an anti-CD80, anti-CD86, anti-MHC class I, or anti-MHC class II Ab and analyzed for expression of surface markers. Bar graphs show the percentages (mean ± SEM of five separate experiments) for each surface molecule on CD11c+ cells. **D**. TNF-α, IL-6, IL-1β, IL-12p70, and IL-10 levels in the culture medium were measured by an ELISA. Data are presented as mean ± SEM (n = 5). **E**. Dot plots of intracellular IL-12p70 and IL-10 expression in CD11c^+^ DCs. *p < 0.05, **p < 0.01, and ***p < 0.001 for treated compared to untreated DCs (CON).

There is little information about the im-munological roles of the Rv2299c protein in the mycobacterial HSP family. We purified the recombinant Rv2299c protein in *Escherichia coli* BL21 to study its immunoreactivity. Purity of Rv2299c was assessed by SDS-PAGE and western blot analysis (Figure 1A). Endotoxin content of prepared Rv2299c was below 15 pg/ml (<0.1 EU/ml) according to an LAL assay. DCs and macrophages play a major role in initiation and activation of the protective immune response to mycobacteria. Therefore, we examined the effect of Rv2299c on macrophage activation or DC maturation. Rv2299c activated both DCs and macrophages, but the increase in the amount of cytokine secretion was more dramatic in the DCs than in the macrophages (data not shown). Endocytic activity as a marker of DC maturation was measured by exposure to dextran-fluorescein isothiocyanate (FITC). Double-positive cells (CD11c^+^ and dextran-FITC-positive) were decreased in number among DCs treated with Rv2299c or lipopolysaccharide (LPS), which served as a positive control, indicating that endocytic activity was reduced and functional maturity was enhanced by this protein (Figure 1B). Furthermore, DCs treated with Rv2299c for 24 hr showed strongly enhanced expression of MHC classes I and II and of costimulatory molecules such as CD80 and CD86 in a dose-dependent manner (Figure 1C). These results suggest that Rv2299c efficiently induces DC maturation.

Some studies have shown that DC-derived cytokines stimulate the polarization of T cells and inflammatory responses [19]. Therefore, next, we tested whether DC maturation by Rv2299c was coupled with the secretion of pro- or anti-inflammatory cytokines. As shown in Figure 1D, Rv2299c significantly stimulated DCs to secrete large amounts of TNF-α, IL-6, and IL-1β, whereas untreated DCs secreted negligible amounts of these cytokines. We then determined the production of IL-12p70 and IL-10, which have an important effect on the development of T-cell immune responses. Unlike LPS, Rv2299c significantly induced the secretion of IL-12p70, but not IL-10 (Figure 1D). Fluorescence-activated cell sorting (FACS) analysis also revealed that DCs treated with Rv2299c showed an increased percentage of IL-12p70-positive cells as compared to untreated DCs, while no change was found in IL-10-positive cells (Figure 1E). Although endotoxin content was monitored all the time during the preparation of the recombinant protein, we further assessed LPS contamination by treatment with proteinase K or heat denaturation, which abrogated the ability of Rv2299c to trigger DC maturation (Supplementary Figure 1). The effects of Rv2299c were not inhibited by polymyxin B treatment, whereas those of LPS were significantly inhibited. Taken together, these results suggest that Rv2299c induces secretion of proinflammatory cytokines from DCs, and these Rv2299c-matured DCs may promote a Th1-type immune response.

### Rv2299c induces DC maturation via the TLR4 pathway

**Figure 2:**
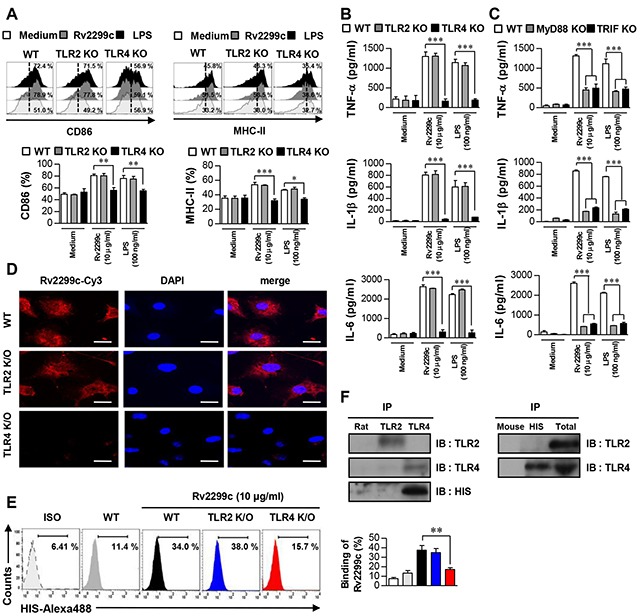
Rv2299c induces DC activation via TLR4 Bone marrow-derived DCs from WT, TLR2^−/−^, and TLR4^−/−^ mice were treated with Rv2299c (10 μg/ml) or LPS (100 ng/ml) for 24 hr. **A**. Expression of CD86 and MHC class II was determined by staining and flow cytometry. Bar graphs show the percentages (mean ± SEM of three separate experiments) for each surface molecule on CD11c+ cells. **B**. and **C**. DCs derived from WT, TLR2^−/−^, or TLR4^−/−^ and WT, MyD88^−/−^, or TRIF^−/−^ mice were stimulated with Rv2299c or LPS for 24 hr and the produced cytokines were determined by an ELISA. **D**. Fluorescence intensity of the anti-His antibody bound to Rv2299c-treated DCs. DCs derived from WT, TLR2^−/−^, and TLR4^−/−^ mice were treated with Rv2299c (10 μg/ml) for 1 hr, fixed, and stained with DAPI and a Cy3-conjugated anti-His antibody (Scale bar: 10 mm). **E**. DCs treated with Rv2299c for 1 hr and stained with an Alexa 488-conjugated anti-His mAb. Representative flow cytometry (left). The percentage of positive cells shown in each panel are also presented as mean ± SEM of three independent experiments (right). ISO: isotype control. **F**. DCs were treated with Rv2299c (10 μg/ml) for 6 hr. The cells were harvested, and cell lysates were immunoprecipitated (IP) with an anti-rat IgG, anti-mouse IgG, anti-His, anti-TLR2, or anti-TLR4 Ab; then proteins were visualized by immunoblotting (IB) with anti-His, anti-TLR2, or anti-TLR4 Abs. Totals are shown as mean total cell lysates (input). All data are expressed as mean ± SD (*n* = 3). One representative data point of three experiments is shown; *p < 0.05, **p < 0.01 and ***p < 0.001 for different treatments compared to Rv2299c- or LPS-treated WT DCs.

Several Mtb components that activate DCs via a Toll-like receptor (TLR) pathway have been identified [20]. Therefore, we determined whether Rv2299c could be recognized by, and act through, TLRs in DCs. The expression of surface molecules (Figure 2A) and proinflammatory-cytokine secretion (Figure 2B) were significantly suppressed in DCs from TLR4^−/−^ mice when compared to DCs from wild-type (WT) or TLR2^−/−^ mice, indicating that Rv2299c may be an agonist of TLR4 in DCs. MyD88 is a common adaptor molecule to all the TLRs, whereas TRIF is essential for TLR4 signaling pathway [21]. Determination of proinflammatory-cytokine production in DCs from WT, MyD88^−/−^, and TRIF-deficient mice suggested that a MyD88- and TRIF-dependent signaling pathway was involved in Rv2299c-induced production of TNF-α, IL-6, and IL-1β (Figure 2C). Next, we tested whether Rv2299c binds to TLR4 in DCs. Confocal microscopy with an Cy3-conjugated anti-His antibody (Ab) revealed that Rv2299c bound preferentially to the surface of WT and TLR2^−/−^ DCs but not TLR4^−/−^ DCs (Figure 2D). The binding between Rv2299c and the TLR4 molecule was also confirmed by FACS analysis (Figure 2E). To further confirm the binding between Rv2299c and TLR4, we performed immunoprecipitation analysis with an anti-TLR2 or anti-TLR4 Ab and an anti-His Ab. We found that Rv2299c binds to TLR4, but not TLR2 (Figure 2F). These findings clearly revealed that Rv2299c induced DC maturation in a TLR4-dependent manner, causing increased expression of cell-surface molecules and proinflammatory cytokines.

### MAPK and NF-κB pathways are involved in Rv2299c-mediated DC maturation

**Figure 3:**
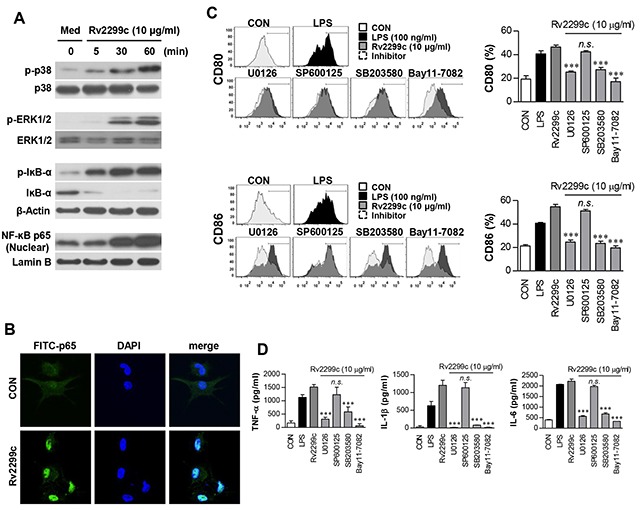
Involvement of the ERK1/2 and p38 but not JNK pathways in Rv2299c protein-induced maturation of DCs **A**. Protein production by DCs treated with Rv2299c for indicated periods was analyzed by immunoblotting using each specific Ab: to phospho-p38 (p-p38), p38, phospho-ERK1/2 (p-ERK1/2), phospho-IκB-a, IκB-a, and p65 NF-κB. **B**. Effects of Rv2299c on cellular localization of the p65 subunit of NF-κB in DCs. DCs were plated in covered glass chamber slides and treated with Rv2299c for 1 hr, and immunoreactivity of the p65 subunit of NF-κB in cells was determined by immunofluorescence. **C**. and **D**. DCs were pretreated with pharmacological inhibitors of p38 (SB203580, 20 μM), ERK1/2 (U0126, 10 μM), JNK (SP600125, 20 μM), Bay11-7082 (5 μM), or DMSO (vehicle control) for 1 hr prior to treatment with 10 mg/ml Rv2299c protein for 24 hr. The expression of CD80 and CD86 was analyzed by flow cytometry (C). Bar graphs show the percentages (mean ± SEM of three separate experiments) for each surface molecule on CD11c^+^ cells. The amounts of TNF-α, IL-6, and IL-1β in the culture medium were measured by ELISAs (D). The mean ± SEM are shown for three independent experiments; *p < 0.05, **p < 0.01, or ***p < 0.001 for inhibitor treatments compared to Rv2299c-treated controls.

MAPKs and NF-κB are important signaling molecules for controlling the maturation of DCs and for secretion of proinflammatory cytokines [22, 23]. We therefore examined the activation of MAPKs and NF-κB in response to Rv2299c. As expected, Rv2299c triggered phosphorylation of p38 and ERK1/2 as well as phosphorylation and degradation of IκB-α in DCs (Figure 3A), and induced significant translocation of p65 from the cytosol to the nucleus (Figure 3B). To confirm the activation of MAPKs and NF-κB in Rv2299c-induced proinflammatory cytokine production and costimulatory molecule expression, DCs were pretreated with a p38 inhibitor (SB203580), an ERK1/2 inhibitor (U0126), a JNK inhibitor (SP600125), or an NF-κB inhibitor (Bay 11-0782) for 1 hr before exposure to Rv2299c. All the pharmacological inhibitors except the JNK inhibitor significantly suppressed Rv2299c-induced expression of the costimulatory molecules on the surface of DCs (Figure 3C) and the production of proinflammatory cytokines (Figure 3D). Based on these findings, we believe that the MAPK and NF-κB signaling pathways are essential for production of proinflammatory cytokines and for expression of DC maturation markers induced by Rv2299c.

### Rv2299c-maturated DCs induce naïve-T-cell proliferation

**Figure 4:**
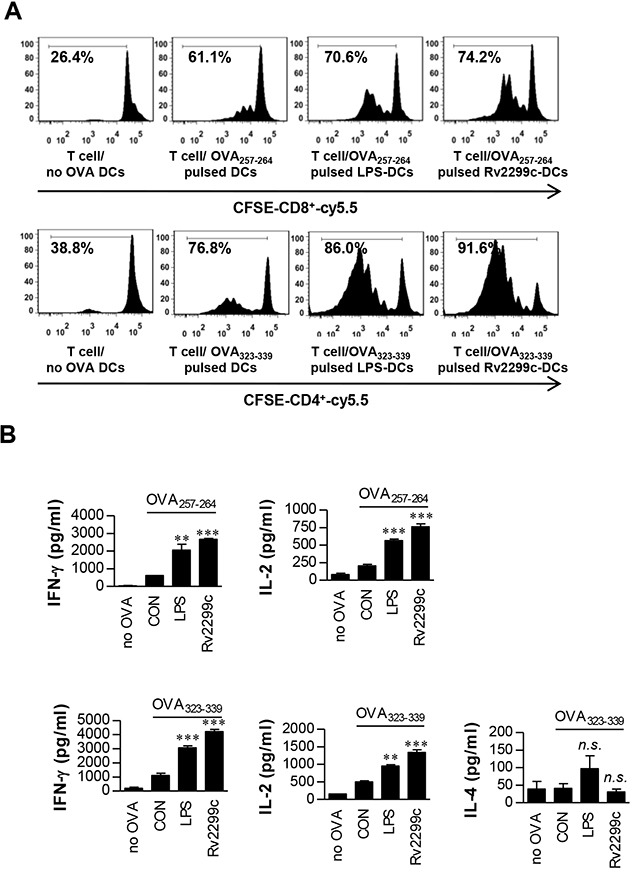
Rv2299c protein-treated DCs induce T-cell proliferation **A**. Transgenic OVA-specific CD8^+^ T cells and transgenic OVA-specific CD4^+^ T cells were isolated, stained with CFSE, and cocultured for 96 hr with DCs treated with Rv2299c (10 μg/ml) or LPS (100 ng/ml), then pulsed with OVA_257–264_ (1 μg/ml) for OVA-specific CD8^+^ T cells or OVA_323–339_ (1 μg/ml) for OVA-specific CD4^+^ T cells, respectively. T cells only and T cells cocultured with untreated DCs served as controls. The proliferation of OT-I^+^ and OT-II^+^ T cells was then assessed by flow cytometry. **B**. The culture supernatants harvested after 24 hr and IFN-γ, IL-2, and IL-4 were assayed by ELISA. The mean ± SEM is shown for three independent experiments; *p < 0.05 for treatments when compared to the appropriate controls (T cell/OVA_257–264_ pulsed DCs or T cell/OVA_323–339_ pulsed DCs). *n.s*.: no significant difference.

To precisely characterize Rv2299c activity on the interaction between DCs and T cells, we performed a syngeneic *in vitro* T-cell proliferation assay using OT-I T cell receptor (TCR) transgenic CD8^+^ T cells and OT-II TCR transgenic CD4^+^ T cells. DCs pulsed with OVA_257–264_ or OVA_323–339_ were cocultured with transgenic CFSE-labeled OVA-specific CD4^+^ or CD8^+^ T cells for 72 hr. Rv2299c- or LPS-treated DCs induced T-cell proliferation to a significantly greater extent as compared to untreated DCs (Figure 4A). Furthermore, naïve CD4^+^ and CD8^+^ T cells primed with Rv2299c-treated DCs produced significantly greater IFN-γ and IL-2 amounts than did those with untreated DCs, whereas IL-4 secretion did not increase regardless of Rv2299c stimulation (Figure 4B). These results suggest that the Rv2299c-treated DCs direct naïve-T-cell proliferation toward a Th1 phenotype.

### Rv2299c-maturated DCs induce expansion of the effector/memory T-cell population

**Figure 5:**
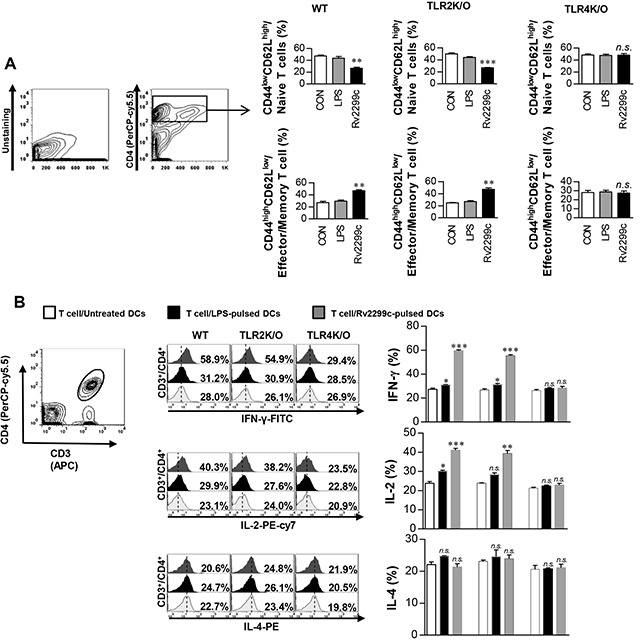
Rv2299c protein-treated DCs induce expansion of effector/memory T-cell population DCs from WT, TLR2^−/−^, and TLR4^−/−^ mice were treated with Rv2299c (10 μg/ml) or LPS (100 ng/ml), and then cocultured for 3 d with T cells from Mtb-infected mice at DC to T cell ratios of 1:10. Splenocytes were stained with anti-CD4, anti-CD62L, and anti-CD44 mAbs. **A**. A histogram is shown for gating of the labeled T cells. Bar graphs show CD62L^low^CD44^high^ T cells or CD62L^high^CD44^low^ T-cell populations among the spleen cells. **B**. Intracellular IFN-γ, IL-2, or IL-4 expression in CD3^+^/CD4^+^ T cells cocultured with untreated DCs, Rv2299c-treated DCs, or LPS-stimulated DCs. The percentages of CD3^+^/CD4^+^ T cells among all T cells are indicated in the top right corner. Mean ± SEM for three independent experiments is shown; **p < 0.01 or ***p < 0.001 for treatments compared to untreated DCs. *n.s*.: no significant difference.

To determine whether Rv2299c-stimulated DCs have an ability to specifically stimulate CD4^+^ from Mtb-infected mice, we analyzed the expression change of CD62L and CD44 on CD4^+^ splenic T cells induced by Rv2299c-treated DCs using flow cytometry. DCs were prepared from the bone marrow of WT, TLR2^−/−^, or TLR4^−/−^ mice and maturated with Rv2299c or LPS. After coculture of the Rv2299c-maturated DCs and syngeneic CD4^+^ T cells from the infected mice, Rv2299c-maturated DCs from WT or TLR2^−/−^ mice but not from TLR4^−/−^ mice caused significantly downregulated CD62L and upregulated CD44 expression in CD4^+^ T cells when compared to control DCs or LPS-treated DCs (Figure 5A). The percentages of CD4-IFN-γ- or CD4-IL-2-positive cells were significantly higher for coculture with Rv2299c-maturated DCs from WT or TLR2^−/−^ mice but not TLR4^−/−^ mice when compared to control DCs or LPS-stimulated DCs (Figure 5B). In addition, the numbers of CD4-IL-4-positive cells were not increased by coculturing with the antigen-stimulated DCs. These data suggest that Rv2299c-maturated DCs induce the expansion of effector/memory T cells and drive Th1 immune responses in a TLR4-dependent manner.

### T cells activated by Rv2299c-maturated DCs inhibit intracellular Mtb growth

**Figure 6:**
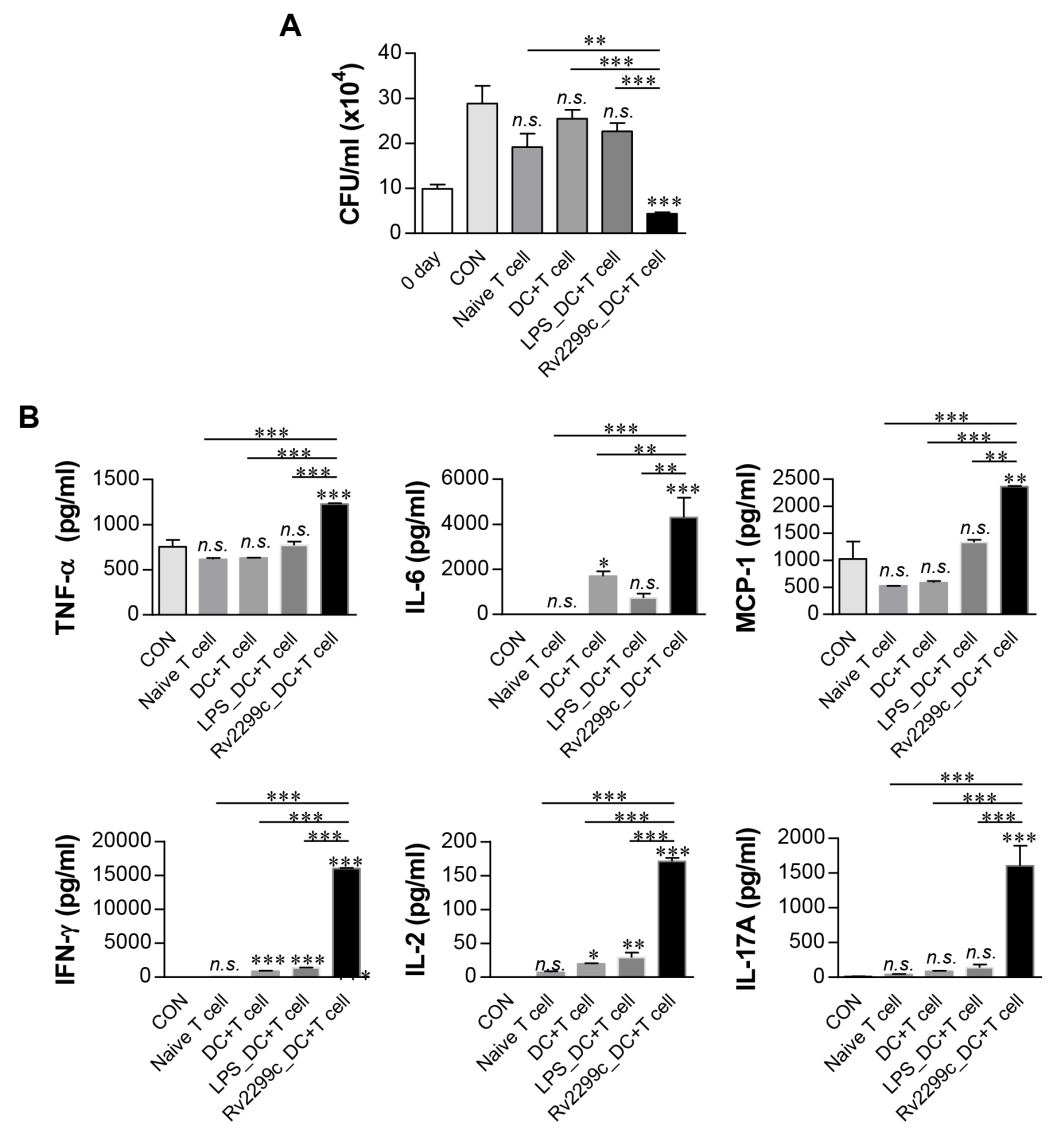
T cells activated by Rv2299c-maturated DCs inhibit intracellular Mtb growth Naïve T cells or T cells activated by unstimulated DCs, LPS-stimulated DCs, or Rv2299c-stimulated DCs at a DC:T cell ratio of 1:10 for 3 d were cocultured with BMDMs infected with Mtb. **A**. Intracellular Mtb growth in BMDMs was determined at time point 0 (0 day) and 3 d after coculturing with T cells or without T cells (control). **B**. The cytokine levels in culture supernatants were measured by ELISA. The data shown are the mean values ± SD (*n* = 3); *p < 0.05, **p < 0.01, or ***p < 0.001 for BMDMs cocultured with T cells compared to control BMDMs. n.s.: no significant difference.

To confirm that Rv2299c-maturated DCs actually play a role in the control of Mtb, we tested whether T cells activated by Rv2299c-maturated DCs could enhance the bactericidal activity of macrophages. Naïve splenic T cells from uninfected mice were activated by coculturing with Rv2299c-maturated DCs for 72 hr, and then added to Mtb-infected bone marrow-derived macrophages (BMDMs). As shown in Figure 6A, the simple addition of unactivated T cells elicited a considerable inhibition of intracellular Mtb growth. Interestingly, T cells activated by Rv2299c-maturated DCs significantly inhibited the Mtb growth as compared to T cells activated by unactivated DCs or LPS-maturated DCs. Proinflammatory cytokines such as IFN-γ and IL-17, which are related to antimycobacterial activity, showed significantly higher expression levels during the addition of T cells activated by Rv2299c-maturated DCs in comparison with that observed with the addition of T cells activated by unactivated DCs or LPS-maturated DCs. These data indicated that inhibition of Mtb growth is induced by the production of these cytokines (Figure 6B). Significant IFN-γ and IL-2 production was induced by addition of T cells activated by unactivated DCs or LPS-maturated DCs as compared to addition of T cells only. These results suggest that Rv2299c induces activation of the T cells with bactericidal activity via DC maturation.

### Fusion of Rv2299c to ESAT-6 enhances the immunoreactivity of ESAT-6

**Figure 7:**
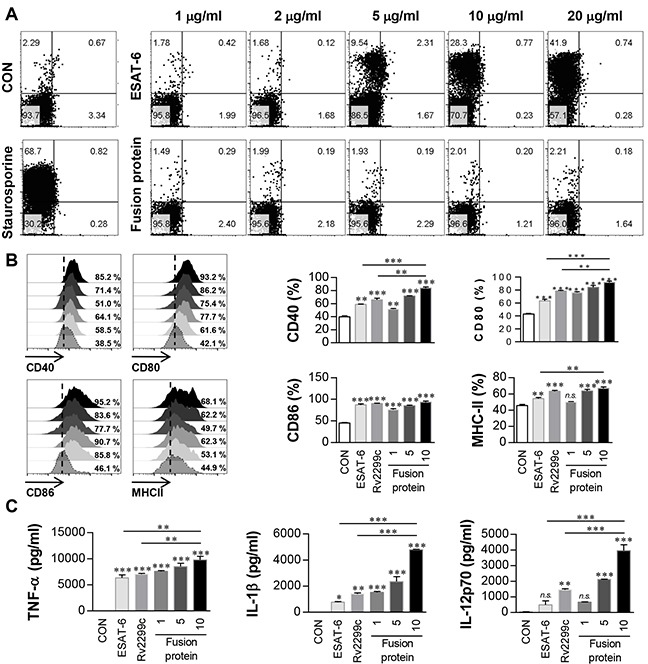
Immunoreactivity of Rv2299c-ESAT-6 fusion protein **A**. BMDCs treated with the indicated concentration of ESAT-6 or Rv2299c-ESAT-6 fusion protein for 24 hr were analyzed by flow cytometry using staining with anti-CD11c, annexin V, and PI. Staurosporine was used as a positive control. The results are representative of three experiments. **B**. BMDCs treated with ESAT-6 (2 μg/ml), Rv2299c (10 μg/ml), or the Rv2299c-ESAT-6 fusion protein (1, 5, 10 μg/ml) for 24 hr were analyzed by two-color flow cytometry. The cells were gated to exclude CD11c cells. DCs were stained with anti-CD40, anti-CD80, anti-CD86, or anti-MHC class II. The results are representative of the results of three experiments. Bar graphs show mean ± SEM (*n* = 5). **C**. The cytokine levels in culture supernatants were measured by ELISA. Data are presented as mean ± SEM (*n* = 5); *p < 0.05, **p < 0.01, and ***p < 0.001 for different treatments compared to untreated control (CON).

**Figure 8:**
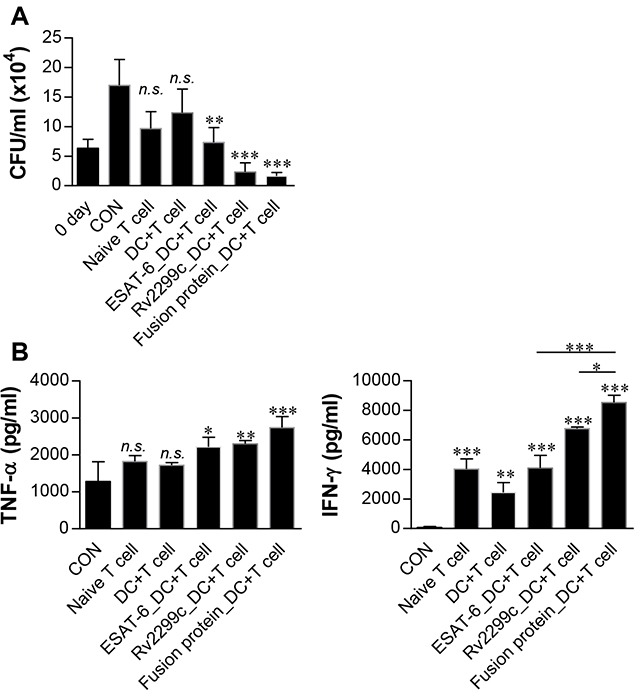
Inhibition of intracellular Mtb growth by T cells activated with Rv2299c-ESAT-6 fusion protein-maturated DCs Naïve T cells or T cells activated by unstimulated DCs, ESAT-6-stimulated DCs, Rv2299c-stimulated DCs, or the fusion protein-stimulated DCs at a DC:T cell ratio of 1:10 for 3 d were cocultured with BMDMs infected with Mtb. Intracellular Mtb growth in BMDMs **A**. and the cytokine levels **B**. were determined as in Figure 6. The data shown are mean values ± SD (*n* = 3); *p < 0.05, **p < 0.01, or ***p < 0.001 for BMDMs cocultured with T cells compared to control BMDMs. *n.s*.: no significant difference.

We hypothesized that a DC-activating protein can enhance the protective immunity of a T-cell-stimulating antigen. To test this notion, ESAT-6, a major T-cell vaccine candidate, was expressed and purified as a protein fused to Rv2299c in *E. coli*, and its immunogenicity was assessed. First, we determined the cytotoxicity of the recombinant Rv2299c-ESAT-6 fusion protein in BMDMs by annexin V and propidium iodide (PI) staining. ESAT-6 showed cellular toxicity at a concentration above 5 μg/ml, but the fusion protein did not exert toxicity at 20 μg/ml (Figure 7A). Therefore, ESAT-6 was used at the concentration of 2 μg/ml in subsequent experiments. Next, we further assessed LPS contamination by treatment with proteinase K or heat denaturation, which abrogated the ability of Rv2299c-ESAT-6 fusion protein to trigger DC maturation (Supplementary Figure 2). The effects of Rv2299c were not inhibited by polymyxin B treatment, whereas those of LPS were significantly inhibited. Expression of costimulatory and MHC molecules (Figure 7B) and production of proinflammatory cytokines such as TNF-α, IL-1β, and IL-12 (Figure 7C) was significantly increased in DCs stimulated with the fusion protein in comparison with a single protein. Next, we also determined bactericidal activity of T cells stimulated by the fusion protein-maturated DCs. As shown in Figure 8A, intracellular Mtb growth was significantly inhibited by addition of T cells activated with ESAT-6-maturated DCs as compared to control BMDMs without any T cells or DCs. Nevertheless, T cells activated by Rv2299c-stimulated DCs or Rv2299c-ESAT-6 fusion protein-maturated DCs significantly inhibited the growth of intracellular Mtb as compared to any other condition. The highest production of proinflammatory cytokines such as IFN-γ and TNF-α was detected when Mtb-infected BMDMs were cocultured with T cells activated by the fusion protein-stimulated DCs (Figure 8B). IL-4 production was not detected in any condition (data not shown).

### The Rv2299c-ESAT-6 fusion protein has significant BCG prime boosting effects against the hypervirulent HN878 strain

**Figure 9:**
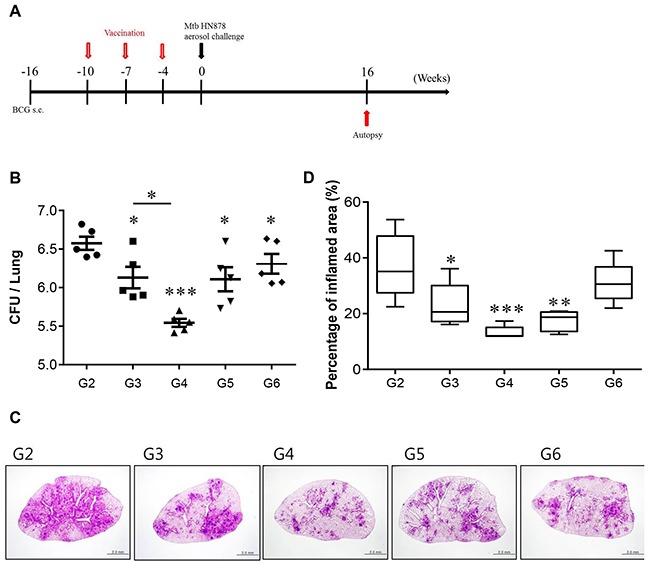
CFU counts for each group and histological analysis of representative lung lobes **A**. A schematic diagram of the experimental design. **B**. Differences in the bacterial burden among the mice immunized with BCG alone, protein alone, or the BCG/Rv2299c-ESAT-6 fusion protein and those treated with the adjuvant control (MPL-DDA alone) at 16 weeks after a challenge with Mtb HN878 are shown (*n* = 5 animals/group). **C**. Lung sections from each immunized mouse were stained with H&E at 16 weeks after challenge with Mtb HN878. **D**. The percentage of the inflamed area from a lung section; *p < 0.05, **p < 0.01, and ***p < 0.001 compared with the MPL/DDA-alone group. Group 1 (G1): infection control, G2: MPL/DDA control, G3: BCG alone, G4: BCG/Rv2299c-ESAT-6 fusion protein- MPL/DDA, G5: the Rv2299c-ESAT-6 fusion protein- MPL/DDA, G6: ESAT-6- MPL/DDA.

Finally, we determined whether the Rv2299c-ESAT-6 fusion protein has a vaccine potential against TB. First, to determine the value of Rv2299c as a vaccine antigen, protective efficacy of Rv2299c alone in prophylactic settings was evaluated against Mtb Erdman. However, vaccination with Rv2299c/MPL-DDA did not confer significant protection against Erdman in terms of reduction of bacterial burden in the lungs and spleen (data not shown). We next demonstrated the level of protection against an Mtb HN878 challenge provided by immunization with the Rv2299c-ESAT-6 fusion protein as a BCG-prime booster. Previous studies showed that BCG vaccination of mice provides less protection against W-Beijing isolates than against Mtb H37Rv over 10 weeks postinfection [24]. Therefore, we tested the vaccine efficacy of the protein in a murine model using the clinical hypervirulent HN878 strain, belonging to the W-Beijing family, as described previously [25]. Four weeks after final vaccination, we challenged the mice with the Mtb HN878 strain and determined the bacterial loads in their lungs 16 weeks postchallenge (Figure 9A). As shown in Figure 9B, BCG only, ESAT-6, and the Rv2299c-ESAT-6 fusion protein (separately) significantly reduced the bacterial loads in the lungs as compared to adjuvant control. There was no significant difference in protective efficacy among these three vaccines, although the ESAT-6 immunized group showed a higher bacterial load and worse pathological findings in the lungs. We also evaluated the ability of the fusion protein to boost the BCG vaccine. Sixteen weeks after infection, the fusion protein-boosted mice had a significantly lower bacterial count than the BCG-vaccinated mice did. Overall, the Rv2299c-ESAT-6 fusion protein showed protective efficacy against an HN878 strain challenge in terms of bacterial count reduction at distant time points. In addition, at 16 weeks postchallenge, the Rv2299c-ESAT-6 fusion protein-immunized group showed significantly reduced lung inflammation as compared with the infection control group (*p* < 0.01; Figure 9C and 9D).

### Antigen-specific Th1 immune responses in mice infected with Mtb HN878

**Figure 10:**
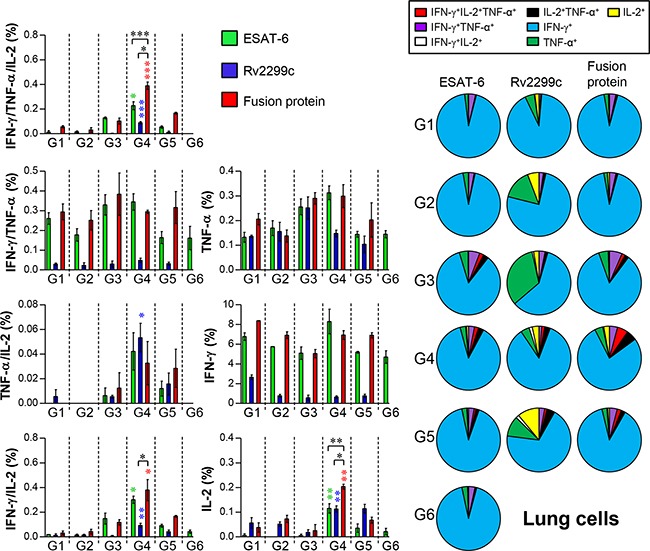
Induction of antigen-specific multifunctional T cells in the lungs of mice after a challenge with Mtb HN878 strain Sixteen weeks postinfection, mice in each group (*n* = 6) were euthanized, and their lung cells (2.0 × 10^6^) were stimulated with each antigen (5 μg/ml) for 12 hr at 37°C in the presence of GolgiStop. The percentage of antigen-specific CD4^+^ T cells producing IFN-γ, TNF-α, and/or IL-2 among the cells isolated from a lung of each group of mice were analyzed by multicolor flow cytometry by gating for CD4^+^ lymphocytes. Pie charts represent the mean frequencies of cells coexpressing IFN-γ, TNF-α, and/or IL-2. Data are presented as mean ± SD from five mice in each group and unpaired *t* test was used to determine statistical significance; differences with *p* < 0.05 were considered statistically significant; *p < 0.05, **p < 0.01, and ***p < 0.001 compared with the MPL-DDA-alone group (unpaired *t* test). Description of each group is shown in the legend of Figure 9.

The Th1-mediated immune response and multifunctional T cells play important roles in protective immunity against Mtb. Thus, we next evaluated the change in the T-cell phenotype induced by Rv2299c-ESAT-6 fusion protein immunization. Postchallenge, spleen and lung cells were stimulated *in vitro* with ESAT-6, Rv2299c, or the fusion protein, and the phenotypes of the responding CD4^+^ T cells were evaluated by multicolor intracellular cytokine staining and flow cytometry (Supplementary Figure 3). Increased numbers of triple-positive CD4^+^ T cells (coexpressing IFN-γ, TNF-α, and IL-2) in the lungs (Figure 10) and spleens (Supplementary Figure 4) were observed in the BCG/Rv2299c-ESAT-6 fusion protein-immunized group (group 4) only (not in other groups). In addition, the Rv2299c-ESAT-6 fusion protein-immunized group showed an increased frequency of double-positive multifunctional CD4^+^ T cells (TNF-α^+^IL-2^+^ CD4^+^ and IFN-γ^+^IL-2^+^ CD4^+^ T cells) and single-positive (IL-2^+^ CD4^+^) T cells in the lungs and spleens postchallenge.

## DISCUSSION

Subunit protein vaccines are potentially useful for BCG replacement or for boosting BCG-induced immune responses [26]. Here, we presented a vaccine strategy designed to improve protective efficacy of TB vaccine candidates. The selection of optimal antigens to be included in a polyprotein vaccine is the most important step for vaccine development. DCs regulate naïve-T-cell polarization and subsequent development of a Th1- or Th2-type response in a draining lymph node. Therefore, a DC-activating protein-based vaccine may enhance protective immunity of other antigens in the formulation. In this study, we found that the Rv2299c protein capable of activating DCs is a promising candidate for TB vaccine design, because the Rv2299c-ESAT-6 fusion protein significantly reduced the bacterial load in lungs after a challenge with the highly virulent Mtb HN878 clinical strain.

The fundamental rationale for the development of TB vaccines designed to elicit T-cell-based immunity is based on the assumption that a strong Th1 immune response specifically directed against Mtb antigens is the primary mechanism for antituberculosis immunity. Antigens that do not elicit Th1 responses uniformly fail to protect against Mtb, but not all proteins that induce robust Th1 responses after vaccination provide considerable protection [27]. The MVA85A vaccine, which elicits a robust Th1 response against Ag85A, does not provide protection on top of that conferred by BCG in humans [28]. It is important to note that a vaccine induces a memory T-cell response and facilitates development of long-term immunity. DCs play a crucial role in effective activation of T cells and induction of memory T-cell responses. It is reported that mice vaccinated with Mtb-infected DCs [29] or Mtb sonicate-pulsed DCs [16] are protected against Mtb infection. These results support that activating DCs *in vivo* is crucial in the development of antimycobacterial vaccine [30]. In this study, to test the hypothesis that DC-activating proteins are viable vaccine candidates, choosing a reliable DC-activating protein was an important research question. First, we selected mycobacterial HSPs because they have the ability to activate immune cells such as macrophages or DCs [31–33] and an adjuvant activity [34]. In the present study, among the mycobacterial HSPs, we found that Rv2299c (HSP90) induces DC maturation through TLR4 signaling and MAPK and NF-κB activation. This activation led to increased expression of costimulatory molecules and secretion of proinflammatory cytokines to promote a Th1-type immune response. It was recently reported that DCs isolated from splenocytes of mice injected with Rv2299c show increased expression of MHC molecules and costimulatory molecules, and tumor antigen-loaded DCs in combination with Rv2299c treatment have a synergistic antitumor immunity effect [35]. These results and our findings suggest that Rv2299c has an adjuvant activity via DC activation *in vivo* and *in vitro*.

It has been reported that several mycobacterial proteins activate DCs to drive a Th1 immune response via the TLR2 [36, 37] or TLR4 pathway [38, 39], or to drive a Th2 immune response via the TLR2 pathway [22], indicating that each antigen has different effects in DCs. Recombinant mycobacterial HSP70 signals through both TLR4 and TLR2, whereas mycobacterial HSP65 signals exclusively through TLR4 to activate immune cells [40]. Human HSP90 elicits biological activities through the TLR4 pathway [41, 42]. The present study showed that Rv2299c, which belongs to the HSP90 family, activates DCs in a TLR4-, MyD88-, and TRIF-dependent manner, which was demonstrated by a pulldown assay and experiments on TLR4 or TLR2 knockout mice.

The subunit vaccines consisting of two to four fused proteins evoke strong protective responses with efficacy that is equivalent to the protection induced by BCG alone [26]. Most people have been vaccinated with BCG in infancy, but the efficacy of this vaccine wanes over time [43], thus boosting the protective immunity induced by BCG may be the most practical strategy. Therefore, we analyzed the protective immune response and efficacy of the fusion protein consisting of Rv2299c and ESAT-6. We selected ESAT-6 as a fusion partner because it is one of the major antigens included in the vaccines currently in clinical trials. ESAT-6 secretion is essential for cell death induction in infected cells [44, 45]. In the present study, Rv2299c-fused ESAT-6 did not induce cell death, thereby suggesting that the fusion protein may lead to efficient DC maturation without cytotoxicity. We observed similar protective efficacy in mice vaccinated with either BCG alone or the fusion protein. It has been suggested that memory T-cell recall responses are not dependent on DCs and other nonprofessional APCs [46, 47]. However, Wakim et al. showed that activation of memory T cells in response to systemic or localized infection is predominantly dependent on DCs [48, 49]. In the present study, the Rv2299c-maturated DCs induced the expansion of effector/memory CD4^+^ T cells from Mtb-infected mice, which indicated that Rv2299c might induce memory T-cell recall responses in BCG-vaccinated subjects. Moreover, Rv2299c incorporation into the fusion protein improved the immunogenicity of ESAT-6, thus pointing towards a BCG-prime boosting effect of the Rv2299c-ESAT-6 fusion protein.

It is reported that BCG vaccination of mice provides less protection against W-Beijing isolates than against Mtb H37Rv [24]. This isolate causes death and extensive lung pathology in infected C57BL/6 mice. Therefore, we assessed the protective effect of the Rv2299c-ESAT-6 fusion protein as a BCG booster against the Mtb HN878 strain, a hypervirulent W-Beijing-lineage strain, in a mouse model. In general, vaccine-induced protection against Mtb infection specifically involves antigen-specific multifunctional IFN-γ^+^ TNF-α^+^ IL-2^+^ and TNF-α^+^ IL-2^+^ CD4^+^ T cells in the lungs [50]. According to these trends, Rv2299c can likely function as a central component of a successful vaccine against Mtb infection. Immunization with the Rv2299c-ESAT-6 fusion, but not with BCG or with the stand-alone protein, induced an Rv2299c-fused ESAT-6-specific multifunctional CD4^+^ T-cell response in lungs. The ability of the vaccine candidate to induce Rv2299c-ESAT-6 fusion-specific multifunctional T-cell responses in the lungs after intramuscular injection is consistent with the observed protective efficacy. In addition, this response induced by immunization with the Rv2299c-ESAT-6 fusion protein was maintained up to 16 weeks postinfection; this result is suggestive of the presence of primed multifunctional memory CD4^+^ T cells and their rapid expansion after the recognition of Mtb infection. This finding underscores the importance of appropriately adjuvanted-vaccine candidates for induction of protective pulmonary immune responses.

In this study, we employed monophosphoryl Lipid A (MPL) as an adjuvant for the Rv2299c subunit vaccine. However, TRIF-based TLR4 activation by MPL [51] may not induce an optimal Th1 immune response. A recent study showed that synergistic interactions between MyD88 and TRIF are required for Th1-cell polarization with a TLR4 agonist adjuvant [52]. Accordingly, activation of MyD88 by Rv2299c and activation of TRIF by MPL may have synergistic interactions to elicit the protective efficacy of the Rv2299c-fused ESAT-6 subunit vaccine by enhancing Th1 immune responses against Mtb. However, for more practical settings, the optimal combination between antigens and adjuvant should be evaluated by considering their immunological features. In addition, mouse immune cells clearly differ from those of humans. For example, previous studies demonstrated that TLR4 agonists barely induce CD8^+^ T cells in humans because although mouse CD8α^+^ DCs express TLR4, their human counterparts (CD141^+^ DCs) do not [53, 54]. In the same context, the contribution of CD8^+^ T cells has recently been emphasized in protective immunity against Mtb infection [55, 56]. Thus, further experiments on the comparison of the adjuvant effect between TLR4- and TLR3-based adjuvants on the vaccine efficacy of the Rv2299c-ESAT-6 fusion should be conducted.

Nevertheless, to the best of our knowledge, the BCG-prime boosting strategy with a subunit vaccine against highly virulent Mtb clinical isolates has not been tested to date. Because various studies showed that BCG vaccination of mice provides less protection against W-Beijing isolates than from Mtb H37Rv isolates [24, 51], a single dose of parental BCG vaccine is unlikely to maintain protective immunity against highly virulent strains. Further research is needed to optimize the boosting dose and timing to improve the efficacy of this BCG-prime boosting strategy. Moreover, to enter clinical testing, further study for *in vivo* toxicity should be investigated. In any case, in this study, DC-activating Rv2299c antigen and ESAT-6-based subunit vaccine was found to exert a durable BCG-prime boosting effect against hypervirulent HN878 in mice. In conclusion, our results suggest that Rv2299c is an excellent candidate for the rational design of an effective multiantigenic TB vaccine.

## MATERIALS AND METHODS

### Ethics statement

All animal studies were performed in accordance with Korean Food and Drug Administration (KFDA) guidelines. The experimental protocols used in this study were reviewed and approved by the Ethics Committee and Institutional Animal Care and Use Committee (Permit Number: 2014-0197-3) of the Laboratory Animal Research Center at Yonsei University College of Medicine (Seoul, Korea) and IACUC (CNU-00284) of animal care at Chungnam National University (Daejeon, Korea).

### Bacterial strains and preparation of *Mycobacterium* spp

Mtb H37Rv (ATCC 27294) and H37Ra (ATCC 25177) were purchased from American Type Culture Collection (ATCC, Manassas, VA), and Mtb HN878 was obtained from the strain collections of the International Tuberculosis Research Center (ITRC, Changwon, Gyeongsangnam-do, South Korea). *M. bovis* BCG (Pasteur strain 1173P2) was kindly provided by Dr. Brosch at the Pasteur Institute (Paris, France). All mycobacteria used in this study were prepared as described previously [25].

### Animals

Specific pathogen-free 5- to 6-week-old female C57BL/6 mice as well as OT-I and OT-II T-cell receptor (TCR) transgenic mice (C57BL/6 background), C57BL/6 (H-2K^b^ and I-A^b^), C57BL/6J TLR2 knockout mice (TLR2^−/−^; B6.129-Tlr2^tm1Kir^/J), and C57BL/10 TLR4 knockout mice (TLR4^−/−^; C57BL/10ScNJ) were purchased from the Jackson Laboratory (Bar Harbor, ME, USA). The mice were maintained under barrier conditions in a BL-3 biohazard animal facility at the Yonsei University Medical Research Center with constant temperature (24°C ± 1°C) and humidity (50% ± 5%). The animals were fed a sterile commercial mouse diet with ad libitum access to water under standardized light-controlled conditions (12 hr light and 12 hr dark periods). The mice were monitored daily, and none of the mice showed any clinical symptoms or illness during this experiment.

### Abs and reagents

Recombinant mouse macrophage colony-stimulating factor (M-CSF), granulocyte-macrophage colony stimulating factor (GM-CSF), and interleukin 4 (IL-4) were purchased from CreaGene (Gyeonggi, Republic of Korea). Fluorescein isothiocyanate (FITC)-annexin V/propidium iodide kits were purchased from R&D Systems (Minneapolis, MN, USA). Dextran-FITC (molecular mass, 40,000 Da) was acquired from Sigma (St. Louis, MO, USA). Lipopolysaccharide (LPS) from *Escherichia coli* O111:B4 was purchased from InvivoGen (San Diego, CA, USA). The endotoxin filter (END-X) and endotoxin removal resin (END-X B15) were acquired from the Associates of Cape Cod (East Falmouth, MA, USA). The OT-I peptide (OVA_257–264_) and OT-II peptide (OVA_323–339_) were synthesized by Peptron (Daejeon, Korea). The anti-phosphorylated-ERK1/2 monoclonal Ab, anti-ERK1/2 monoclonal Ab, anti-phosphorylated p38 monoclonal Ab, anti-p38 monoclonal Ab, anti-NF-κB (p65) polyclonal Ab, anti-phosphorylated IκB-α monoclonal Ab, anti-IκB-α monoclonal Ab, anti-lamin B polyclonal Ab, and anti-β-actin polyclonal Ab were acquired from Cell Signaling Technology (Danvers, MA, USA). The HRP-conjugated anti-mouse IgG Ab and HRP-conjugated anti-rabbit Ab were obtained from Calbiochem (San Diego, CA, USA), and the anti-β-actin mAb (AC-15) was purchased from Sigma. The FITC-conjugated mAbs against CD11c, p65, IFN-γ, and CD62L, the APC-conjugated mAbs against IL-12p70, IL-10, and CD3, the PerCP-Cy5.5-conjugated mAb against CD4 and CD8, the APC-Cy7-conjugated mAb against CD8^+^, the phycoerythrin (PE)-conjugated mAbs against CD80, CD86, MHC class I, MHC class II, IFN-γ, and CD44, the PE-Cy7-conjugated mAbs against CD11c and IL-2, and the eFluor® 450-conjugated mAb against CD3e were purchased from eBioscience (San Diego, CA, USA). The phycoerythrin (PE)-conjugated rat anti-IgG1, rat anti-IgG2a, and rat anti-IgG2b, the APC-conjugated rat anti-IgG2a and rat anti-IgG1, the FITC-conjugated rat anti-IgG2b, and the PE-Cy7-conjugated mouse anti-IgG1 and rat anti-IgG2b Abs were obtained from eBioscience. These antibodies were used as isotype controls. TNF-α, IL-1β, IFN-γ, IL-2, IL-4, IL-6, IL-10, and IL-12p70 ELISA kits were obtained from eBioscience.

### Expression and purification of recombinant proteins

To produce a recombinant Rv2299c protein, the corresponding gene was amplified by PCR using *M. tuberculosis* H37Rv ATCC27294 genomic DNA as a template and the following primers: *Rv2299c* forward, 5′- CATAT GAACGCCCATGTCGAGCAGTTG-3′, and reverse, 5′-GAATTCGGCAAGGTACGCGCGAGACGTTC-3′; *ESAT-6* forward, 5′-AAGCTTATGACAGAGCAGCAGTGGAAT-3′, and reverse, 5′-CTCGAGTGCGAACATCCCAGTGACGTT-3′. The PCR product of *Rv2299c* was digested with *Nde*I and *EcoR*I, and ESAT-6 was cut with *Hind*III and *Xho*I. The products were inserted into the pET22b (+) vector (Novagen, Madison, WI, USA), and the resultant plasmids were sequenced. The recombinant plasmids were transfected into *E. coli* BL21 cells by heat-shock for 1 min at 42°C. To produce a recombinant fusion protein, the PCR products of Rv2299c were inserted into previously produced ESAT-6-containing pET22b (+) vector. The recombinant protein was prepared as previously described [52].

### Cell culture

Murine bone marrow-derived DCs were generated, cultured and purified as recently described [52]. Bone marrow-derived macrophages (BMDMs) were prepared using recombinant M-CSF, as previously described [53]. Briefly, bone marrow cells isolated from C57BL/6 mice were lysed with red blood cell (RBC)-lysing buffer (ammonium chloride 4.15 g/500 ml, 0.01 M Tris-HCl buffer pH 7.5 ± 2) and washed with the RPMI 1640 medium. The obtained cells were plated in six-well culture plates (10^6^ cells/ml, 3 ml/well) and cultured at 37°C in the presence of 5% CO_2_ in RPMI 1640 media supplemented with 100 unit/ml penicillin/streptomycin (Lonza), 10% of fetal bovine serum (Lonza), 50 μM mercaptoethanol (Lonza), 0.1 mM nonessential amino acids (Lonza), 1 mM sodium pyruvate (Sigma), 20 ng/ml GM-CSF, and 10 ng/ml IL-4 (BMDC) or 20 ng/ml M-CSF (BMDM).

### Cytotoxicity analysis

Cytotoxicity analysis was conducted using an Annexin V/propidium iodide (PI) staining kit (BD Biosciences). The cells were stained with FITC-conjugated Annexin V and PI. Analysis of the stained cells was performed on a FACSCanto II with FACSDiva, and the results were analyzed using the FlowJo software (Tree Star, Ashland, OR, USA).

### Analysis of the expression of surface molecules by flow cytometry

On day 6, BMDCs were harvested, washed with PBS, and resuspended in FACS washing buffer (2% FBS and 0.1% sodium azide in PBS). The cells were preincubated with 0.5% BSA in PBS for 30 min and washed with PBS. The cells were stained with PE-conjugated anti-H-2Kb (MHC class I), anti-I-Ab (MHC class II), anti-CD80, and anti-CD86 along with FITC-conjugated anti-CD11c antibodies for 45 min at 4°C. The cells were washed three times with PBS and resuspended in 500 μl of PBS. The fluorescence was measured by flow cytometry and the data were analyzed using CellQuest data analysis software.

### Antigen uptake ability of BMDCs by Rv2299c

BMDCs (2 × 10^5^ cell) were equilibrated at 37°C or 4°C for 45 min and then pulsed with fluorescein-conjugated dextran at a concentration of 1 mg/ml. Cold staining buffer was added to stop the reaction. The cells were washed three times, stained with PE-conjugated anti-CD11c antibodies, and then analyzed with the FACSCanto. Nonspecific binding of dextran to DCs was determined by incubation of DCs with FITC-conjugated dextran at 4°C, and the resulting background value was subtracted from the specific binding values.

### Confirmation of LPS decontamination for Rv2299c

To confirm that the maturation of DCs induced by Rv2299c was not due to contaminating endotoxins or LPS in the protein preparations, a pretreatment with Polymyxin B (PmB) (Sigma), heat-denaturation, and digestion with proteinase K (Sigma) were performed. DCs were preincubated with 50 μg/ml PmB for 1 hr at room temperature prior to treatment with 100 ng/ml LPS and 10 μg/ml Rv2299c. For heat-denaturation, LPS or Rv2299c was incubated at 100°C for 1 hr. For digestion with proteinase K, LPS or Rv2299c were digested for 1 hr at 37°C with soluble proteinase K at the concentration of 10 μg/ml followed by heating for 15 min at 100°C to deactivate the enzyme, and subsequently added to BMDCs cultures. After 24 hr, TNF-α and IL-6 levels in the supernatant of BMDCs were analyzed using an ELISA.

### Confocal laser scanning microscopy

DCs were plated overnight on poly-L-lysine-coated glass coverslips. After treatment with Rv2299c, the cells were fixed in 4% paraformaldehyde, permeabilized in 0.1% Triton X-100, and then blocked with 2% bovine serum albumin (BSA) in PBS containing 0.1% Tween 20 (PBS/T) for 2 hr before incubation with 2% BSA in PBS/T containing an anti-Rv2299c antibody for 2 hr at room temperature. After a wash with PBS/T, the cells were reincubated with a Cy-3-conjugated secondary antibody in the dark room for 1 hr, and then were stained with 1 μg/ml of DAPI for 10 min at room temperature. Cell morphology and fluorescence intensity were examined using a confocal laser scanning microscope (Zeiss LSM510 Meta; Carl Zeiss Ltd, Welwyn Garden City, UK). Images were acquired using the LSM510 Meta software and processed using the LSM image examiner.

### Immunoprecipitation

DCs (10^7^) were incubated with 10 μg/ml Rv2299c for 6 hr, and cell pellets were lysed with lysis buffer (10 mM Tris-HCl [pH 7.4], 1% NP-40, 0.25% sodium deoxycholate, 150 mM NaCl, 1 mM EDTA, 1 mM PMSF, 1 μg/ml each aprotinin, leupeptin, and pepstatin, 1 mM Na_3_VO_4_, and 1 mM NaF). To prevent nonspecific binding, the cell lysates were precleared by adding 50 μl of normal serum (Santa Cruz) and 100 μl of 50% protein A or G Sepharose bead slurry (Invitrogen, Carlsbad, CA) to 1 mg of cell lysates. After 2-hr incubation at 4°C, the mixture of beads and cell lysates was centrifuged at 10,000 × *g* for 5 min at 4°C, and the supernatant was collected for the subsequent experiment. Rv2299c (His)-, TLR2-, and TLR4-associated proteins were immunoprecipitated by incubation with protein A or G Sepharose for 24 hr at 4°C after incubation with an anti-rat IgG Ab as a control Ab for anti-TLR2 and TLR4, an anti-mouse IgG Ab as a control Ab for the anti-Rv2299c (His) Ab for 1 hr at 4°C. The beads were harvested, washed and boiled in 5× sample buffer for 5 min. The proteins were separated by SDS-PAGE in a 10% gel followed by transfer of the proteins to a polyvinylidene difluoride membrane (Millipore). The membranes were further probed with anti-TLR2, anti-TLR4, and anti-His Abs as indicated.

### Immunoblotting analysis

After stimulation with 10 μg/ml Rv2299c, DCs were lysed in 100 ml of lysis buffer containing 50 μM Tris-HCl (pH 7.5), 150 mM NaCl, 1% Triton X-100, 1 mM EDTA, 50 mM NaF, 30 mM Na_4_PO_7_, 1 mM phenylmethanesulfonyl fluoride, 2 μg/ml aprotinin, and 1 mM pervanadate. Whole-cell lysate samples were resolved on SDS-polyacrylamide gels and then transferred onto a nitrocellulose membrane. The membranes were blocked in 5% skim milk and incubated with a primary Ab for 2 hr, followed by incubation with HRP-conjugated secondary Abs for 1 hr at room temperature. Epitopes on target proteins including MAPKs and NF-κB recognized specifically by Abs were visualized by means of the ECL Advance Kit (GE Healthcare, Little Chalfont, UK).

### Nuclear extract preparation

Nuclear extracts from cells were prepared as follows. DCs were treated with 100 μl of lysis buffer (10 mM HEPES [pH 7.9], 10 mM KCl, 0.1 mM EDTA, 0.5% Nonidet P-40, 1 mM dithiothreitol [DTT], 0.5 mM PMSF) on ice for 10 min. After centrifugation at 4000 rpm for 5 min, the pellet was resuspended in 100 μl of extraction buffer (20 mM HEPES [pH 7.9], 400 mM NaCl, 1 mM EDTA, 1 mM DTT, 1 mM PMSF) and incubated on ice for 30 min. After centrifugation at 12,000 rpm for 10 min, the supernatant containing nuclear extracts was collected and stored at -80°C until analysis.

### Treatment of DCs with pharmacological inhibitors for analysis of signaling pathways

All the pharmacological inhibitors were purchased from Calbiochem. Dimethyl sulfoxide (Sigma) was added to cultures at 0.1% (vol./vol.) as a solvent control. DCs were washed with PBS and pretreated with inhibitors in the RPMI 1640 medium containing glutamine for 1 hr prior to treatment with Rv2299c for 24 hr. Inhibitors were used at following concentrations: U0126 (10 μM), SB203580 (20 μM), SP600125 (10 μM), and Bay11-7082 (20 μM). In all experiments with the inhibitors, a tested concentration was used after careful titration experiments assessing the viability of the DCs using an MTT assay.

### An *in vitro* T-cell proliferation assay

Responder T cells, which participate in naïve-T-cell reactions, were isolated using a MACS column (Miltenyi Biotec) from total mononuclear cells extracted from BALB/c mice. Both OVA-specific CD8^+^ and CD4^+^ T cells, responders, were obtained from splenocytes of OT-1 and OT-2 mice, respectively. These T cells were stained with 1 μM CFSE (Invitrogen) as previously described [54]. DCs (2 × 10^5^ cells per well) treated with the OVA peptide in the presence of 10 μg/ml of Rv2299c for 24 hr were cocultured with CFSE-stained CD8^+^ and CD4^+^ T cells (2 × 10^6^) at DC:T cell ratios of 1:10. On day 3 or 4 of coculture, each T cell batch was stained with PerCP-Cy5.5-conjugated anti-CD4^+^ mAb, PE-Cy5-conjugated anti-CD4^+^ mAb, PE-Cy5-conjugated anti-CD8^+^ mAb, Alexa 647-conjugated anti-CCR3 mAb, or PE-conjugated anti-CXCR3 mAb and analyzed by flow cytometry. The supernatants were harvested and assayed for the production of IFN-γ, IL-2, and IL-4 by ELISAs.

### Analysis of the activation of effector/memory T cells

As explained above, responder T cells, which participate in allogeneic T-cell reactions, were isolated using a MACS column (Miltenyi Biotec) from total mononuclear cells extracted from *M. tuberculosis*-infected BALB/c mice. Staining with an APC-conjugated anti-CD3 mAb (BD Biosciences) revealed that the preparation consisted mainly of CD3^+^ cells (>95%). DCs (2 × 10^5^ cells per well) isolated from wild-type (WT), TLR2^−/−^, and TLR4^−/−^ C57BL/6 mice were treated with Rv2299c for 24 hr followed by extensive washing and were cocultured with 2 × 10^6^ responder allogeneic T cells (*M. tuberculosis*-infected T cells) at DC:T cell ratios of 1:10. On 4 d of coculture, the cells were stained with PerCP-Cy5.5-conjugated anti-CD4^+^ mAb, PerCP-Cy5.5-conjugated anti-CD8^+^ mAb, FITC-conjugated anti-CD62L mAb, and PE-conjugated anti-CD44 mAb, and analyzed by means of a flow cytometer.

### Quantification of cytokines

A sandwich enzyme-linked immunosorbent assay (ELISA) was used for detecting IL-6, IL-1β, TNF-α, IFN-γ, IL-4, IL-2, IL-12p70, and IL-10 in culture supernatants as described previously [52]. Single cells prepared from the lungs of the immunized or infected mice were stimulated with PPD (2 μg/ml) or antigen-specific CD4 or CD8 T cell peptides (2 μg/ml) for 24 hr at 37°C. The IFN-γ cytokine levels in the culture supernatant were measured using a commercial ELISA kit (eBioscience).

### Intracellular cytokine assays

Cells were first blocked with 10% (vol./vol.) normal goat serum for 15 min at 4°C and then stained with FITC-conjugated CD11c^+^ antibody for 30 min at 4°C. Cells stained with the appropriate isotype-matched immunoglobulin (Ig) served as negative controls. The cells were fixed and permeabilized with the Cytofix/Cytoperm kit (BD Biosciences). Intracellular IL-12p70, IL-10, IL-2, IL-4, and IFN-γ were detected with fluorescein-conjugated antibodies (BD Biosciences) in a permeation buffer. For intracellular cytokine staining, single-cell suspensions from immunized animals (2 × 10^6^ cells) were stimulated with each antigen (5 μg/ml) for 12 hr at 37°C in the presence of GolgiStop (BD Biosciences). The cells were first blocked with Fc Block (anti-CD16/32) for 15 min at 4°C and then stained with BV421-conjugated anti-CD3, PerCp-Cy5.5-conjugated anti-CD4, and FITC-conjugated anti-CD62L antibodies for 30 min at 4°C. These cells were fixed and permeabilized with the Cytofix/Cytoperm kit (BD Biosciences). Intracellular TNF-α, IL-2, and IFN-γ were detected using APC-conjugated anti-TNF-α, PE-Cy7-conjugated anti-IL-2, and PE-conjugated anti-IFN-γ antibodies in a permeation buffer. All antibodies were purchased from eBioscience (San Diego, CA) unless otherwise stated. The cells were analyzed on a FACSverse flow cytometer using the commercially available software program FlowJo (Treestar, Inc., San Carlos, CA).

### Measurement of intracellular Mtb growth in macrophages

Adherent BMDMs (2 × 10^5^ cells/well) were washed twice in PBS and infected in triplicate with Mtb at 2 × 10^5^ bacilli/well. Tubercle bacilli and macrophages were incubated for 4 hr. Then, the infected BMDMs were treated with amikacin (200 μg/ml) for 2 hr. After 2 hr, monolayers were washed to remove extracellular bacilli, and this time point was considered day 0. Next, a previously prepared mixture was added to each well, and the plate was incubated for 3 days. The mixture was antigen-activated DCs cocultured with CD4^+^ T cells at a DC:T cell ratio of 1:10 for 3 days. DC-activating antigens were LPS (100 ng/ml) and Rv2299c (10 μg/ml). The number of ingested and internalized Mtb within the BMDM was calculated by lysing the infected cells from one of the wells in distilled water. The Tubercle bacilli counts of the inoculum were then checked by serial dilution and plating on 7H10 agar with 10% Middlebrook OADC supplement (Difco, Detroit, MI). The plates were incubated at 37°C for 3 weeks. At the end of the 3 weeks, plates were taken out and colony forming units (CFUs) were calculated from the number of colonies of Mtb.

### Vaccination and a challenge in mice

For vaccination, mice were immunized with BCG Pasteur 1173P2 via subcutaneous injection (2.0 × 10^5^ CFUs/mouse) first, then after 6 weeks post-BCG immunization, each subunit vaccine (2 μg) was given three times at 3-weeks intervals with dimethyldioctadecylammonium (DDA) liposomes (50 μg/injection) containing monophosphoryl lipid-A (MPL, 5 μg/injection) (MPL-DDA). Four weeks after the final immunization, the immunized mice were aerogenically challenged with the Mtb HN878 strain as previously described [25, 55]. Briefly, mice were exposed to a predetermined dose of H37Rv or HN878 for 60 min in the inhalation chamber of an airborne infection apparatus (Glas-Col, Terre Haute, IN, USA) to expose the mice to approximately 200 CFUs of viable Mtb. At 16 weeks postchallenge, spleen and lung cells were harvested from each group, and the frequencies of multifunctional T cells and T-cell subtypes were assessed using flow cytometry.

### Bacterial counts and histopathological analysis

Sixteen weeks after the HN878 challenge, six to seven mice per group were euthanized by CO_2_ asphyxiation, and the lungs and spleens were homogenized. The number of viable bacteria was determined by plating serial dilutions of the organ (left lung or half spleen) homogenates onto Middlebrook 7H11 agar (Difco Laboratories, Detroit, MI) supplemented with 10% OADC (Difco Laboratories), amphotericin B (Sigma-Aldrich, St. Louis, MO) and 2 μg/ml 2-thiophenecarboxylic acid hydrazide (Sigma-Aldrich). Colonies were counted after 4 weeks of incubation at 37°C. For the histopathological analysis, the superior lobes of the right lung were stained with H&E and assessed for the severity of inflammation. The level of inflammation in the lungs was evaluated using the ImageJ software (National Institutes of Health, Bethesda, MD), as described previously [56]. In addition, the inflammatory responses were assessed based on lesion size and constitution of immune cells. The data on CFUs and assessment of lung inflammation are reported as the median log_10_ CFU ± interquartile range (IQR).

### Statistical analysis

All the experiments were repeated at least three times with consistent results. The levels of significance for comparison between samples were determined by Tukey's multiple comparison test distribution using statistical software (GraphPad Prism Software, version 4.03; GraphPad Software, San Diego, CA). The data in the graphs are expressed as the mean ± SEM. Differences with each value of *p < 0.05, **p < 0.01, or ***p < 0.001 were considered statistically significant.

## SUPPLEMENTARY FIGURES


