# Supplementary material for: Rv2299c, a novel dendritic cell-activating antigen of Mycobacterium tuberculosis, fused-ESAT-6 subunit vaccine confers improved and durable protection against the hypervirulent strain HN878 in mice
Source: Oncotarget. 2017 Feb 10;8(12):19947–67. doi: 10.18632/oncotarget.15256 (PMC5386736; doi:10.18632/oncotarget.15256)
Supplement: Supplementary file 1 [file oncotarget-08-19947-s001.pdf]

Rv2299c, a novel dendritic cell-activating antigen of *Mycobacterium tuberculosis*, fused-ESAT-6 subunit vaccine confers improved and durable protection against the hypervirulent strain HN878 in mice

SUPPLEMENTARY FIGURES

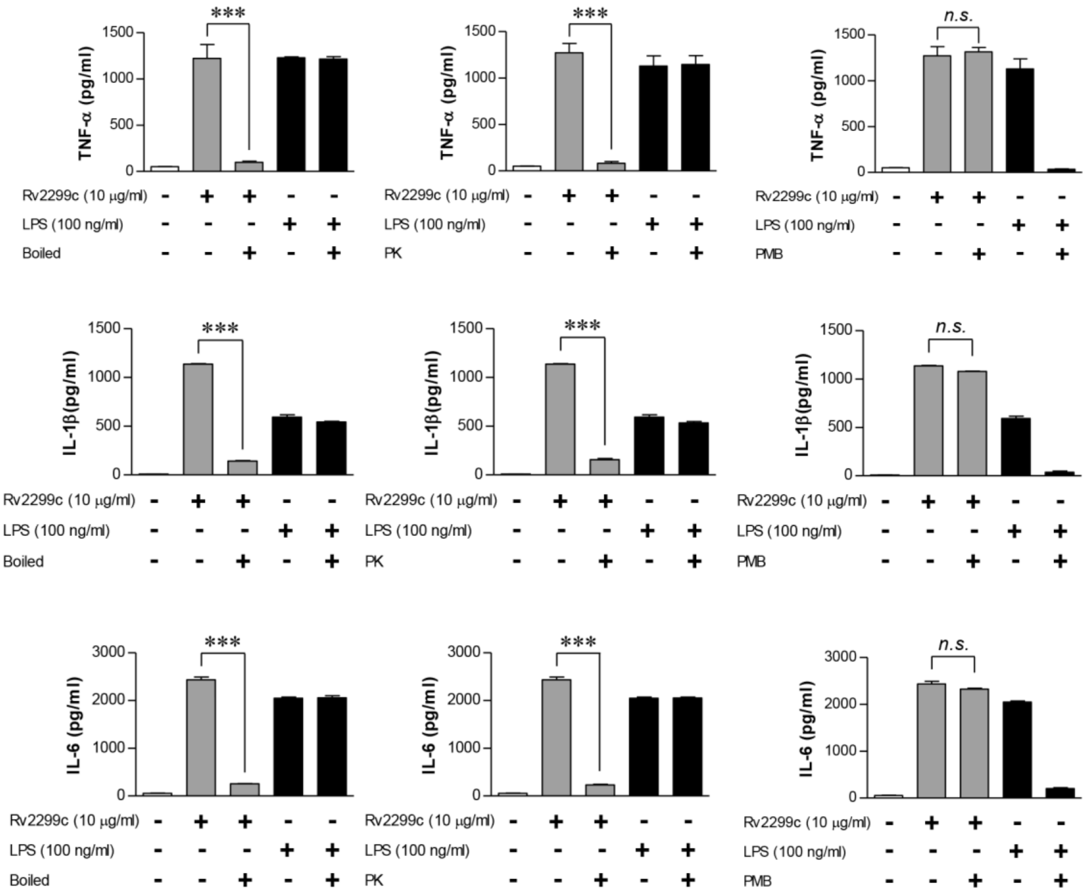

Supplementary Figure 1: Confirmation of endotoxin decontamination of the purified Rv2299c. DCs were stimulated with Rv2299c denatured by boiling for 30 min at 100°C or digested with proteinase K (PK, 10 μg/mL) for 1 hr at 37°C or DCs were pretreated with polymyxin B (50 μg/mL) for 1 hr prior to stimulation of the DCs. LPS (100 ng/ml) was used as control. After 24 hr, the quantities of TNF-α, IL-1β, and IL-6 in the culture medium were measured by ELISAs. All data are expressed as mean ± SD ($n = 3$) and statistical significance (*$p < 0.05$, **$p < 0.01$, or ***$p < 0.001$) is indicated for treatments compared to the controls, whereas treatments that showed no significant effect are indicated as *n.s*.

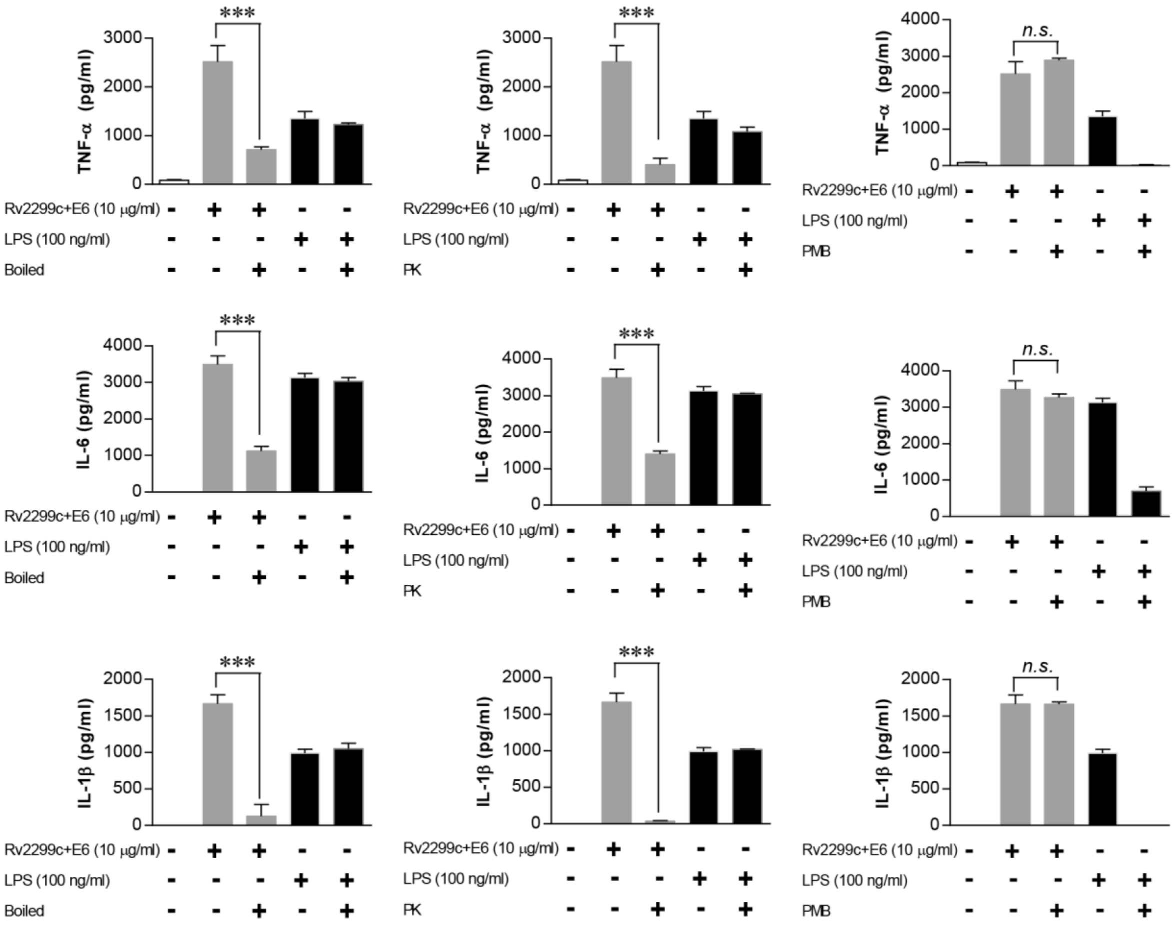

Supplementary Figure 2: Confirmation of endotoxin decontamination of the purified Rv2299c-ESAT-6 fusion protein. DCs were stimulated with Rv2299c-ESAT-6 fusion protein denatured by boiling for 30 min at 100°C or digested with proteinase K (PK, 10 μg/mL) for 1 hr at 37°C or DCs were pretreated with polymyxin B (50 μg/mL) for 1 hr prior to stimulation of the DCs. LPS (100 ng/ml) was used as control. After 24 hr, the quantities of TNF-α, IL-1β, and IL-6 in the culture medium were measured by ELISAs. All data are expressed as mean ± SD ($n = 3$) and statistical significance (*$p < 0.05$, **$p < 0.01$, or ***$p < 0.001$) is indicated for treatments compared to the controls, whereas treatments that showed no significant effect are indicated as *n.s.*

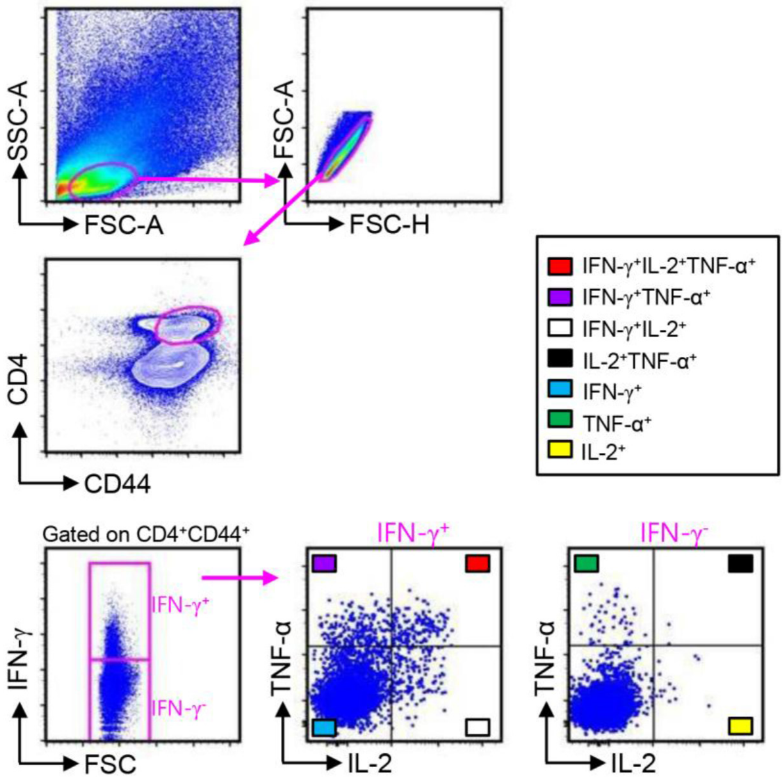

Supplementary Figure 3: The strategy for gating multifunctional CD4⁺ T cells. Spleen and lung cells from immunized mice were stimulated with each antigen (5 μg/ml) for 12 hr in the presence of GolgiStop. Fusion protein-stimulated cells were identified by intracellular cytokine staining based on CD3 and CD4 expression and were further gated for CD44⁺ cells. The percentages of cells expressing all three cytokines (IFN-γ, TNF-α, and IL-2), two of these three cytokines, or one of these three cytokines in each group are depicted in Figure 10.

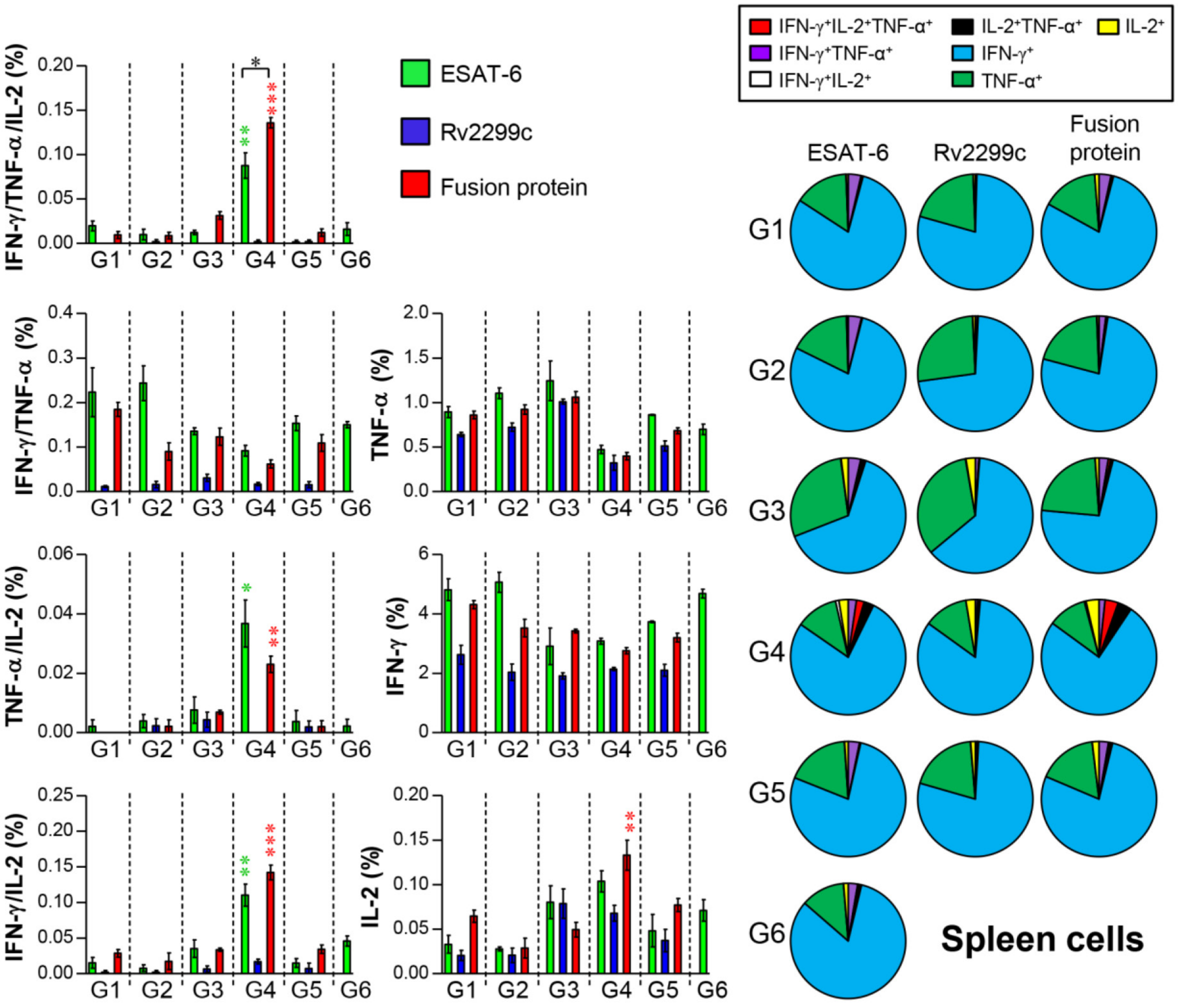

Supplementary Figure 4: Induction of antigen-specific multifunctional T cells in the spleen of mice after challenge with Mtb HN878 strain. Sixteen weeks postinfection, mice in each group (n = 6) were euthanized, and their spleen cells (2.0×10^6 cells) were stimulated with each antigen (5 μg/ml) and analyzed as in the legend of Figure 10. Data are presented as mean ± SD from five mice in each group and the unpaired t test was used to determine significance. A difference with $p < 0.05$ was considered statistically significant; *$p < 0.05$, **$p < 0.01$, and ***$p < 0.001$ compared with the MPL-DDA-alone group (unpaired t test). Description of each group is shown in the legend of Figure 9.
